# Intestinal perforation secondary to intestinal Burkitt lymphoma

**DOI:** 10.1016/j.ijscr.2021.01.085

**Published:** 2021-01-25

**Authors:** Yuji Takayama, Masaaki Saito, Kosuke Ichida, Yuta Muto, Akira Tanaka, Toshiki Rikiyama

**Affiliations:** a Department of Surgery, Jichi Medical University Saitama Medical Center, 1-chome-847 Amanuma-cho, Omiya-ku, Saitama City, Saitama, 330-8503, Japan; b Department of Pathology, Jichi Medical University Saitama Medical Center, 1-chome-847 Amanuma-cho, Omiya-ku, Saitama City, Saitama, 330-8503, Japan

**Keywords:** BL, Burkitt lymphoma, NCCN, National Comprehensive Cancer Network, Burkitt lymphoma, Small intestine, Perforation

## Abstract

**Introduction and importance:**

Small intestinal perforation in patients with Burkitt lymphoma is extremely rare. We present the first report of such a case.

**Case presentation:**

A 53-year-old woman was admitted with abdominal pain and vomiting. Abdominal examination revealed rigidity and tenderness in the upper abdomen.

Computed tomography scan showed thickening of the wall of the jejunum, intra-abdominal free gas, and ascites; the patient was diagnosed with small intestinal perforation, and underwent emergency surgery on the same day. Laparoscopic findings were a 50 mm jejunal perforation and perforation in the transverse mesocolon. A partial jejunal resection of the perforated area, partial transverse colectomy, temporary colostomy, and intra-abdominal drainage were performed. Histological examination showed diffuse infiltration of medium-sized atypical lymphocytes in the perforated area, exhibiting a “starry sky” appearance.

Immunostaining results showed that the atypical lymphocytes were CD20 and virtually 100% Ki-67 positive, and CD56, CD30, and EBER negative. The lesion was identified as Burkitt lymphoma (BL). The postoperative course was favorable. On postoperative day 18 the patient began chemotherapy through the hematology department. Currently, the patient is in remission.

**Clinical discussion:**

The majority of the malignant lymphomas occurring in the digestive tract are identified in the stomach; over 90% are B-cell lymphomas and mucosa-associated lymphoid tissue lymphoma Nakamura et al. BL originating from the small intestine accounts for only about 9%.

**Conclusion:**

The incidence of BL in the small intestine is low. Pretreatment BL can lead to bowel perforation. Prompt involvement of the hemato-oncologist after definitive diagnosis, and commencing chemotherapy as early as possible after surgery, are thought to improve prognosis.

## Highlights

• We experienced a case of small intestinal perforation caused by intestinal Burkitt lymphoma.
• Pretreatment BL may present with bowel perforation.
• This is the first report of such a case.

## 1 Introduction

Malignant lymphomas account for 30%–40% of all malignant tumors in the small intestine, and are a frequently encountered condition [2]. The majority of small intestinal lymphomas are B-cell malignant lymphomas, and the incidence of Burkitt lymphoma (BL) is infrequent (approximately 9%) [1]. It is very difficult to diagnose patients with asymptomatic BL. Some reports presented cases of incidental diagnosis of BL in the small intestine while investigating cases of intussusception [[3], [4], [5], [6], [7], [8], [9], [10], [11]]. However, cases where BL was diagnosed due to small intestine perforations are extremely rare.

We report a case of acute generalized peritonitis due to BL perforation in the small intestine which required life-saving emergent surgery. The aim of this report is to clarify that Burkitt lymphoma can cause small bowel perforation.

The work in this case has been reported in line with the SCARE criteria [12].

## 2 Presentation of case

A 53-year-old woman was brought to the hospital in an ambulance with a 1-month history of recurrent abdominal pain and vomiting. The abdominal pain and frequent vomiting usually occurred after eating. She was diagnosed 1 year prior to presentation with left-sided breast cancer. There is no relevant family or medication history. The patient was admitted for medical evaluation at our facility. Physical examination revealed a weight of 46 kg, height 154 cm, blood pressure 110/62 mmHg, temperature 38.5 °C, and a pulse rate of 90/min. She had generalized tenderness with board like rigidity of the abdomen.

**Fig. 1:**
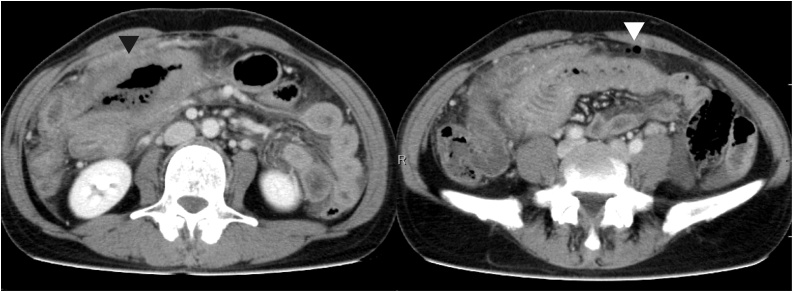
An abdominal computed tomography scan showing thickening of the proximal jejunum wall and the presence of free air in the peritoneal cavity, near the jejunum.

Blood analysis revealed a white blood cell count of 6440/μL, hemoglobin level of 13.4 g/dL, platelet count of 22.2 × 10^4^/μL, C-reactive protein level of 0.82 mg/dL, and lactate dehydrogenase level of 703 U/L. Abdominal computed tomography scans revealed peripheral wall thickening of the jejunum, small amounts of free gas in the abdominal cavity, and ascites (Fig. 1).

**Fig. 2:**
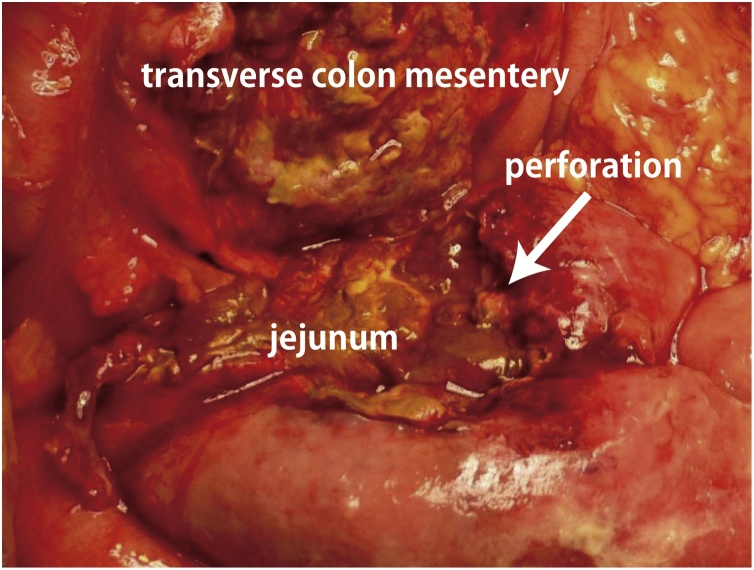
Operative findings demonstrating perforation of the proximal jejunum and penetration of the mesentery of the transverse colon.

A diagnosis of acute generalized peritonitis secondary to small intestinal perforation was made, and an emergency laparotomy was performed. The operation was performed by the first author, who had over 5 years of specialized surgical experiences. A midline incision was made from the umbilicus to the lower abdomen. Cloudy yellow ascitic fluid was noted in the abdominal cavity. The transverse mesocolon adhered firmly to the jejunum. (Fig. 2). A perforated area of the jejunum measuring about 50 mm in diameter was confirmed when the jejunum was freed. Partial jejunal resection along with partial transverse colectomy, jejunostomy, and peritoneal lavage were performed.

**Fig. 3:**
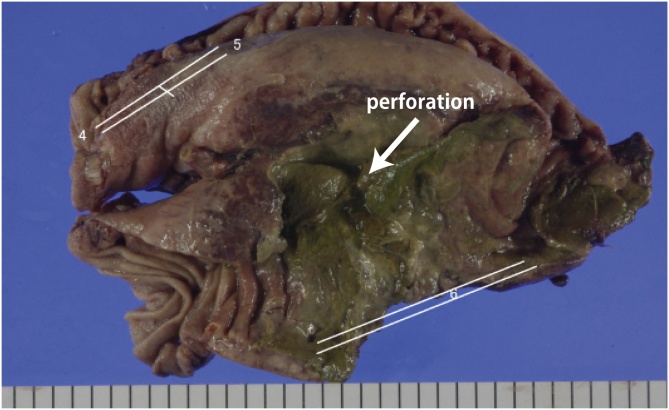
Surgical specimen showing a 50 mm perforation in the proximal jejunum.

The tissue surrounding the perforated area (50 mm) observed in the jejunum was necrosed. Inflammatory changes were observed in the mesentery of the small intestine and the transverse mesocolon near the perforated area (Fig. 3).

**Fig. 4:**
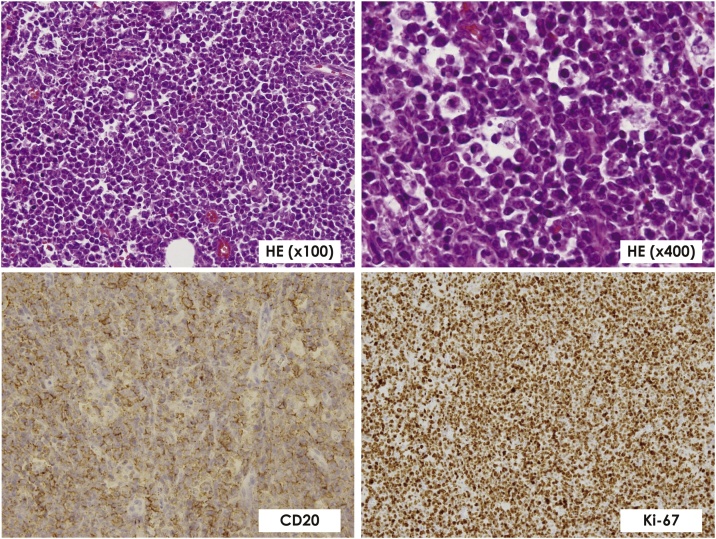
Histopathological findings confirming Burkitt lymphoma with lymphocytes staining positive for CD20 and Ki-67. Ki-67 is positive almost 100% and we diagnosed Burkitt lymphoma.

Histological examination of samples from the perforated area of the jejunum, the mesentery of the small intestine, and the transverse mesocolon, revealed a large number of diffuse medium-sized atypical lymphocyte infiltrates and histiocytes with phagocytosed nuclear fragments, and a “starry sky” appearance. Immunostaining showed that the atypical lymphocytes were CD20 positive, and CD56, CD30, and EBER negative. CD3 and CD5 were positive only for a small number of intervening lymphocytes, and CD20 and Ki-67 staining was positive in almost 100% of the cells (Fig. 4). A diagnosis of BL was made based on these findings.

She had an uneventful postsurgical recovery period and on postoperative day 18 was transferred to the hematology department to commence chemotherapy. To date, the patient has been followed-up for five months and is currently in remission.

## 3 Discussion

BL is a B-cell lymphoma, a form of non-Hodgkin’s lymphoma. It is characterized by aggressive growth and rapid development of clinical features. BL accounts for 1–2% of all malignant lymphomas [13] and is frequently seen in children and young adults [14]. The majority of malignant lymphomas occurring in the digestive tract are identified in the stomach, with reports indicating that over 90% are B-cell lymphomas and mucosa-associated lymphoid tissue lymphoma. BL originating from the small intestine accounts for only about 9% [1].

BL in the small intestine is often characterized by abdominal pain, nausea, constipation, and an abdominal mass [15]. BL frequently occurs in the ileocecal region, the peritoneum, and in retroperitoneal lymph nodes.

A pathognomonic finding is the “starry sky” appearance, characterized by diffuse proliferation of homogeneous cells. These cells are approximately the same size as that of the nuclei of the histiocytes (or slightly smaller) and exhibit prominent macrophages with phagocytose nuclear fragments. The tendency of these cells to proliferate is extremely high, and Ki-67 staining has been reported to be over 95% positive. Some chromosomal translocations, such as t(8;14), t(2;8), and t(8;22) are frequently seen in BL, and most cases exhibit c-MYC gene abnormalities in the eighth chromosome.

The diagnostic criteria of malignant lymphomas in the digestive tract have not been established. However, Dawson et al., suggested the following five diagnostic criteria: no superficial lymph node swelling, no distinct swelling of mediastinal lymph nodes, no abnormalities in the peripheral blood results, mainly gastrointestinal lesions with and metastasis limited to the regional lymph nodes, and no metastases in the liver or spleen [16,17].According to the NCCN (National Comprehensive Cancer Network) guidelines (2013, ver. 2), CODOX-M/IVAC[CPA(cyclophosphamide), DXR(doxorubicin), VCR(vincristine), MTX(methotrexate / ifosfamide, Ara-C(cytarabine), are recommended as the initial treatment regimen for BL etoposide]. Therapies ± rituximab, hyper-CVAD (CPA, VCR, dexamethasone, DXR) therapy + rituximab, dose-adjusted-EPOCH therapy + rituximab, etc. are described, but the superiority or inferiority of each is not described and remains unknown [18].

Multidrug chemotherapy centered on cyclophosphamide hydrate is reported to have an extremely poor prognosis with an average survival time of 5.6 months; however, recent reports have indicated that, in adults, multidrug treatment has resulted in remission in 75%–90% of patients [18]. There are also some reports wherein remission was achieved with chemotherapy and intestinal resection was avoided. Chemotherapy is thought to be associated with a high risk of tumor necrosis and subsequent gastrointestinal perforation. It has also been reported that cases of BL associated with perforation have a poorer prognosis [19]; therefore, performing surgery in order to avoid perforation may improve the prognosis.

Our case satisfied all of the five diagnostic criteria for malignant lymphomas of the digestive tract, as proposed by Dawson et al. Histopathological tests revealed the characteristic “starry sky” appearance, and Ki-67 staining was positive for almost 100% of the lymphocytes; therefore a diagnosis of BL was made. Histopathological findings of the excised bowel also showed severe infiltration of diffuse medium-sized atypical lymphocytes in the jejunal wall around the perforated area, and the primary site of the tumor was diagnosed as the jejunum. The postoperative course was favorable, and the patient was transferred to the hematology department, where she continued to receive chemotherapy and remained in remission as of the 5-month follow-up.

A search of the terms “Burkitt’s/Burkitt lymphoma”, “perforation”, and “small intestine” in PubMed from 2000 to 2020 showed a number of cases where gastrointestinal perforation occurred during chemotherapy. However, to our knowledge, there are no reports of small intestine perforation due to BL of the digestive tract before treatment, and our report seems to be the first of such a case.

In cases of small intestinal perforation in adults, emergency surgery should be conducted as early as possible to avoid life-threatening complications. Remission can be achieved using chemotherapy for a number of gastrointestinal diseases, such as small bowel cancer, gastrointestinal stromal tumor, Crohn's disease, and even aggressive malignancies with an extremely poor natural course, such as BL.

## 4 Conclusion

The incidence of BL in the small intestine is low. Prompt involvement of the hemato-oncologist after a definitive diagnosis is made, and commencing chemotherapy as early as possible after surgery, are thought to influence prognosis.

## Declaration of Competing Interest

All authors declare that there is no conflict of interest.

## Funding

This research did not receive any specific grant from any funding agency in the public, commercial, or not-for-profit sectors.

## Ethical approval

The institutional ethics committee considers that ethical approval is not necessary for a case report.

## Consent

Written informed consent was obtained from the patient for publication of this case report and accompanying images. A copy of the written consent is available for review by the Editor-in-Chief of this journal on request.

## Author contribution

Yuji Takayama, Masaaki Saito: conceptualization, investigation, data curation, writing and reviewing.

Kosuke Ichida, Yuta Muto: conceptualization, investigation, data curation, reviewing.

Akira Tanaka, Toshiki Rikiyama: conceptualization, data curation, writing, reviewing.

## Registration of research studies

Researchregistry6002.

## Guarantor

Masaaki Saito, the corresponding author of this paper.

## Provenance and peer review

Not commissioned, externally peer-reviewed.
